# Analysis of Processing Impact on Raspberries Based on Broad-Spectrum Metabolomics

**DOI:** 10.3390/metabo15070435

**Published:** 2025-06-26

**Authors:** Xiaoge Wang, Qiyuan Liao, Fan Wang, Xuelin Rui, Yushan Liu, Rui Wang

**Affiliations:** 1 Department of Pharmacy, Anhui College of Traditional Chinese Medicine, Wuhu 241000, China; 11lqy19791117@ahzyygz.edu.cn (Q.L.); 11wf19850104@ahzyygz.edu.cn (F.W.); 11rxl19970115@ahzyygz.edu.cn (X.R.); 11lys19891228@ahzyygz.edu.cn (Y.L.); 2 Wuhu Modern Technology Research and Development Center of Chinese Herbal Medicine and Functional Food, Wuhu 241002, China; 3 Dali University, Dali 671000, China; 13611528427@163.com

**Keywords:** raspberries, diabetic nephropathy, MAPK signaling

## Abstract

**Objective:** Our objective was to explore the regulatory mechanism of salt processing on the metabolome of the raspberry and its potential efficacy against diabetic nephropathy (DN), providing metabolomic and network pharmacological evidence for the scientific connotation of traditional Chinese medicine processing. **Methods:** Ultra-high-performance liquid chromatography–tandem mass spectrometry (UHPLC-MS/MS)-based metabolomics was used to compare the metabolic profiles between raw and salt-processed raspberries. Network pharmacology was applied to screen the common targets of the active components in the salt-processed raspberry and DN-related pathways, followed by in vitro cell experiments to validate the regulation of the MAPK signaling pathway. **Results:** The metabolomic analysis identified 80 differentially expressed metabolites, among which 13 key components (VIP ≥ 1, FC ≥ 2) were significantly altered, including enriched flavonoids (e.g., luteolin-7-O-glucoside), triterpenoid saponins (Raspberryides H/F), and phenolic acids (ellagic acid). The network pharmacology revealed that the salt-processed raspberries regulated the DN-related pathways through 122 common targets, with the core nodes focusing on the signaling molecules (e.g., AKT1, EGFR) involved in the MAPK signaling pathway and apoptosis regulation. The in vitro experiments confirmed that the salt-processed raspberry extract (160–640 μg/mL) significantly inhibited the phosphorylation levels of p38/ERK/JNK in high-glucose-induced renal cells. **Conclusions:** This study firstly combines metabolomics and network pharmacology to reveal the regulatory mechanism of salt processing on the active components of raspberries. The salt-processing technology enhanced the inhibitory effect of raspberries on the MAPK signaling pathway, thereby ameliorating the progression of DN. These findings provide scientific support for establishing a metabolomics-based quality control system for traditional Chinese medicine processing. The current findings are primarily based on in vitro models, and in vivo validation using DN animal models is essential to confirm the therapeutic efficacy and safety of salt-processed raspberries.

## 1. Introduction

*Raspberry* (Rosaceae, Rubus), a traditional Chinese medicinal herb with both medicinal and dietary values, belongs to the genus Rubus of the family Rosaceae. It has health benefits such as nourishing the kidneys, consolidating essence, reducing urination, nourishing the liver, and improving eyesight [1]. It is also a commonly used astringent medicine in clinical practice, often employed to treat conditions such as kidney deficiency, nocturnal emissions, urinary frequency, erectile dysfunction, liver and kidney insufficiency, and visual disturbances [2]. *Raspberry* first appeared in the “Shennong Bencao Jing” under the name “Penglei” [3]. The 2020 edition of the “Pharmacopoeia of the People’s Republic of China” includes the dried fruit of the East China Raspberry (*Raspberry* chingii Hu) as the source variety of raspberries [4].

At present, the main processing methods of raspberries include wine-processed raspberries and salt-processed *Raspberry*. Different processing methods lead to variations in the composition and efficacy of raspberries [5]. Research has shown that the ellagic acid content in raspberries increases after salt processing, enhancing their kidney-tonifying and essence-consolidating effects and significantly improving the physiological indicators in a kidney yang-deficiency polyuria rat model [6]. However, the flavonoid content in raspberries decreases after salt processing. Some researchers have also found that four different processing methods have varying effects on the ellagic acid content in raspberries [7]. Among them, the raspberries processed by drying, rehydration fermentation, and then re-drying had the highest ellagic acid content, far exceeding the standards set by the Pharmacopoeia of the People’s Republic of China. The ellagic acid content of the sun-dried raspberries met the pharmacopoeia requirements, while the content in the raspberries dried at 45 °C and those blanched in boiling water followed by sun-drying failed to meet the standards.

Pharmacodynamic studies have shown that salt processing of raspberries can enhance the material basis for kidney-tonifying, essence-consolidating, yang-invigorating, and urine-constraining effects [8]. Experiments have revealed that salt-processed raspberries significantly increased aldosterone levels in a kidney yang-deficiency polyuria rat model induced by adenine, improved the kidney and testicular indices, and exhibited effects superior to those of unprocessed raspberries. Compared with the unprocessed group, the salt-processed *Raspberry* group significantly enhanced the effects of increasing serum cAMP and reducing cGMP in rats [9].

In recent years, research has been conducted on the chemical components and key bioactive substances of raspberries [10,11]. However, the metabolic changes during the processing of raspberries remain unclear. This situation not only hinders the optimization, simplification, and acceleration of *Raspberry* processing but also limits the efficient utilization of bioactive substances, thereby increasing the difficulty of developing high-quality products. Plant metabolomics is an efficient multi-target technology used to study various small-molecule metabolites in plants and their dynamic changes at specific stages. It provides a pathway for the comprehensive characterization of chemical components across different plant species and parts. In recent years, plant metabolomics based on liquid chromatography–mass spectrometry (LC-MS) has gained widespread attention in the field of plant research and has been widely applied in the study of traditional Chinese medicine [12,13,14].

Therefore, the main purpose of this study is to comprehensively analyze the differential metabolites between salt-processed and raw *Raspberry* using plant metabolomics and systematically explore the mechanism of salt-processed *Raspberry* in treating diabetic nephropathy through network pharmacology so as to provide a scientific basis for optimizing the processing technology of raspberries.

## 2. Materials and Methods

### 2.1. Materials

The materials used were Ultra Performance Liquid Chromatography (UPLC) (Sciex, Marlborough, MA, USA) and tandem mass spectrometry (MS/MS) (Sciex, Marlborough, MA, USA) with electrospray ionization (ESI) and an Agilent SB-C18 chromatography column (2.1 mm × 100 mm, 1.8 μm) (Agilent Technologies, Santa Clara, CA, USA).

### 2.2. Sample Preparation

We took the clean raspberries, mixed them with salt water, and let them soak for 2 h. When the salt water was absorbed, we put them in a cage and steamed for 2 h until they were thoroughly cooked. Then, we took them out, cooled them down and dried them. (For every 100 kg of clean raspberries, we used 2 kg of salt) [15]. The samples were placed in a freeze dryer for vacuum freeze-drying. Then, they were ground into a powder using a grinder (30 Hz, 1.5 min). A total of 50 mg of the sample powder was weighed and added to 1.2 mL of −20 °C pre-cooled 70% methanol–water internal standard extraction solution. During the extraction process, the sample was vortexed once every 30 min, for a total of 6 times with each vortex lasting 30 s, and stored overnight at 4 °C. After centrifugation (12,000 r·min^−1^, 3 min), the supernatant was separated and filtered through a microporous membrane (pore size: 0.22 μm, ANPEL, Shanghai, China) and stored in a vial for subsequent UPLC-MS analysis. The above samples were repeated 3 times.

### 2.3. Chromatographic Conditions

Mobile phase A consisted of ultrapure water (with 0.1% formic acid), while mobile phase B was acetonitrile (with 0.1% formic acid). The elution gradient was as follows: 0–9 min, 5–95% B; 9–10 min, 95% B; 10–11.1 min, 95–5% B; 11.1–14 min, 5% B. The flow rate was set at 0.35 mL·min^−1^; the column temperature was maintained at 40 °C; and the injection volume was 2 μL.

### 2.4. Mass Spectrometry Conditions

The ESI source running parameters were as follows: source temperature, 550 °C; ion spray voltage, 5500 V (positive ion mode)/−4500 V (negative ion mode); and ion source gas I (GS I), gas II (GS II), and curtain gas (CUR) set to 50, 60, and 25 psi, respectively (1 psi ≈ 6.895 kPa). The collision-induced ionization parameters were set to high. QQQ scanning used the MRM mode, with the collision gas (nitrogen) set to medium. The declustering potential (DP) and collision energy (CE) for each MRM ion pair were optimized. Specific MRM ion pairs were monitored during each elution period of the metabolites.

### 2.5. Metabolite Analysis

Qualitative analysis of the metabolites was based on the secondary spectra and fragmentation patterns reported in the literature [12], establishing a database called MWDB (Metware Database). During the analysis, we removed the adduct form signals of the K^+^, Na^+^, and NH_4_^+^ adducts. Quantitative analysis of the metabolites was conducted using the multi-reaction monitoring (MRM) method of triple-quadrupole mass spectrometry. First, the precursor ions of the target substances were screened; these precursor ions were induced to fragmenting in a collision chamber, forming many fragment ions. Specific characteristic fragment ions were selected through filtration by the triple-quadrupole method, which excluded interferences and improved quantitative accuracy and reproducibility. The determination of the chemical components (qualitative analysis) primarily relied on the compound’s precise mass number, Q1; retention time, RT; and secondary mass spectrometry, MS2. The Q1/RT/MS2 data from the sample were matched against the local database’s Q1/RT/MS2 data, and the substances were classified into qualitative levels, Level1, Level2, and Level3, based on the matching scores. The higher-confidence Level1 and Level2 levels were selected. The peak areas of the mass spectrometry peaks were integrated, and corrections were made for the mass spectrometry peaks of the same metabolite in different samples to achieve more precise quantification and better reproducibility [16,17].

### 2.6. Network Pharmacology

#### 2.6.1. Collection of Bioactive Components and Diabetic Nephropathy-Related Targets

The differentially identified components (mentioned above) were selected as research subjects. The SDF (Structure Data File) formats of these compounds were downloaded from the PubChem database and imported into the SwissTargetPrediction (24 January 2025) database to predict the potential targets of the active components. The results from both databases were merged, and duplicate targets were removed to obtain the final set of bioactive component-related targets.

#### 2.6.2. Predicting Potential Targets and Constructing the Protein Interaction (PPI) Network

Using the Venny 2.1 online tool to draw a Venn diagram, we identified the intersection targets of the active ingredients and diseases as potential therapeutic targets for diabetic nephropathy. To further explore the direct and indirect regulatory relationships between the potential targets, we imported these targets into the STRING database to construct a PPI network. We set “Homo sapieZC,” with a minimum required interaction score of ≥0.9, and hid the free proteins. We saved the results in TSV format and imported them into the Cytoscape 3.6.0 software. We used the CytoNCA plugin to perform topological analysis, including Average Shortest Path Length (Average Shortest Path Length), Closeness Centrality (Closeness Centrality, CC), Eccentricity (Eccentricity), Degree (Degree, D), Betweenness Centrality (Betweenness Centrality, BC), Neighborhood Connectivity (Neighborhood Connectivity, NC), and Number of Directed Edges (Number Of Directed Edges). We analyzed the key target points of raspberries in treating diabetic nephropathy through the protein network map.

#### 2.6.3. GO Function and KEGG Pathway Enrichment Analysis

We imported the intersection targets of the drugs and diseases into the DAVID database for GO functional annotation and KEGG pathway enrichment analysis. We analyzed the biological functions of the core targets from aspects such as biological processes (BP, biological process), cellular components (CC, cellular component), and molecular functions (MF, molecular function). We sorted the enriched pathways based on *p*-values and “Count” values (the number of potential gene targets in each pathway) to screen for key functional pathways.

### 2.7. Cell Experiments

#### 2.7.1. Cell Culture and Subculture

Based on previous literature research methods [18,19,20], the HepG2 cells were cultured under normal conditions at 37 °C, 95% humidity, and a 5% CO_2_ concentration using a medium containing high concentrations of glucose and insulin, with the addition of 10% FBS and antibiotics/antifungal agents. After two days of normal culturing, the cells were washed with sterile PBS and digested with an appropriate amount of trypsin to confirm cell dissociation before stopping the digestion. The cell suspension was then diluted and distributed into new culture dishes for continued cultivation.

#### 2.7.2. Establishment of Glucose-Induced Cell Model

We selected HepG2 cells in the logarithmic growth phase and spread them into a 96-well plate, with a density of about 6000 cells per well. We allowed them to fully adhere before setting them aside. Then, we starved the HepG2 cells in the medium for 12 h. We discarded the supernatant, added 100 μL of incomplete medium (1% BSA, 1% Hepes 1 M, 5.5 mM glucose), and incubated for 4 h to synchronize all HepG2 cells. In the model group, we added a final concentration of 30 mM glucose solution and stimulated it for 24 h.

#### 2.7.3. CCK-8 Method Used to Detect the Effects of Different Concentrations of Raspberries on Cell Activity

The HepG2 cells were seeded in a 96-well plate. After the cells adhered to the walls, different concentrations of raspberry solution (10, 20, 40, 80, 160, 320, 640, and 1280 µg/mL) were added for treatment, and the cells were cultured for 24–48 h. Subsequently, 10 µL of CCK-8 reagent was added to each well, and the cells were incubated for an additional 1–4 h. The OD450 values of each well were measured using a microplate reader.

#### 2.7.4. Western Blot Analysis

After the treatment according to the experimental groups, the protein concentrations were quantified using the BCA assay. For electrophoresis, 40 μg of protein per lane was loaded. The proteins were initially resolved at 50 V until the samples migrated from the stacking gel into the separating gel, forming a straight line. The voltage was then increased to 100 V, and the electrophoresis was stopped when the dye front reached approximately 1 cm from the bottom of the gel. The proteins were transferred to PVDF membranes using a wet transfer system. The membranes were blocked in 5% non-fat milk diluted in 1× TBST for 2 h at room temperature. The primary antibodies (anti-p-p38, p-ERK, p-JNK, p38, ERK, and JNK) were incubated overnight at 4 °C, followed by incubation with fluorescence-conjugated secondary antibodies (all were repeated three times). After washing, the membranes were imaged using a fluorescence imaging system, and the band intensities were quantified with ImageJ 1.41 software (National Institutes of Health, Bethesda, MD, USA).

## 3. Results

### 3.1. Metabolomic Analysis of Raw and Salted Raspberry Products

**Figure 1:**
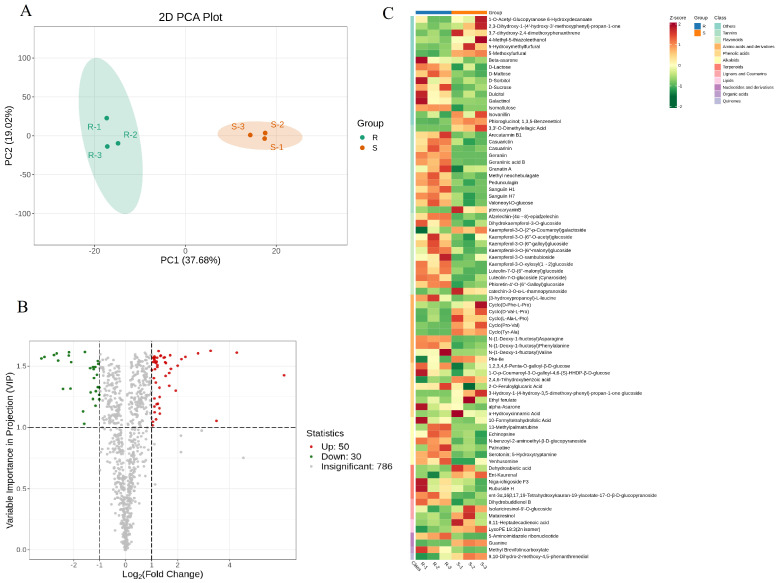
(**A**) PCA; (**B**) volcano; and (**C**) heatmap (R: raw; S: salt).

**Figure 2:**
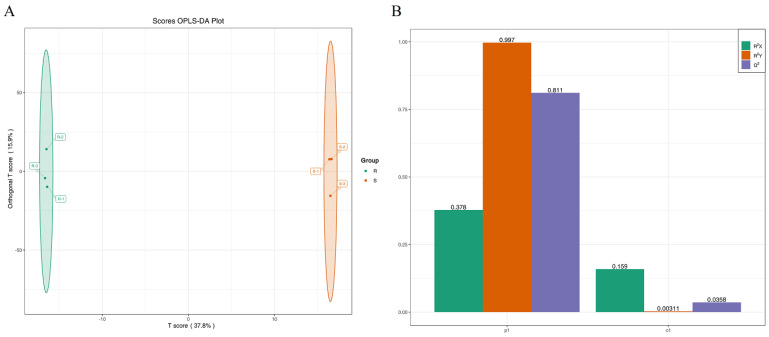
(**A**) OPLS-DA score plot of differential metabolites and (**B**) OPLS-DA model validation plot (R: raw; S: salt).

Using ultra-high-performance liquid chromatography–tandem mass spectrometry (UHPLC-MS/MS), an overlay of the total ion current (TIC) spectra from the mixed quality-control (QC) samples was obtained. The results of the TIC spectra for the QC samples in both the positive and negative ion detection modes showed a high degree of curve overlap (Figure S1). This indicates that the retention times and peak intensities were consistent, demonstrating good signal stability when the same sample was analyzed by mass spectrometry at different times, and that the data results are reliable. Metabolite characterization was conducted through peak-to-peak ratio analysis, leading to the screening of 80 metabolites (detailed in Supplementary Table S1). The raw Raspberry chingii (unprocessed) and salt-processed Raspberry chingii exhibited high similarities in chemical composition yet demonstrated significant quantitative differences in their specific constituents. Systematic studies utilizing chemical markers to differentiate these two variants remain scarce. Principal component analysis (PCA) was applied to the quality control (QC) data to elucidate the metabolomic separation trends and inter-group variability. As shown in Figure 1A, the first two principal components cumulatively accounted for 56.88% of the total variance (PC1 = 37.68%, PC2 = 19.02%). Among these, 50 ions exhibited significant upregulation while 30 showed downregulation, with all ions identified based on retention time (correlation heatmap and volcano plot displayed in Figure 1B and Figure 1C, respectively). The distinct separation between the raw and salt-processed samples in the PCA model confirmed the efficacy of this method for their discrimination. To investigate the contribution of the differential metabolites to the sample discrimination, supervised orthogonal partial least squares–discriminant analysis (OPLS-DA) was performed on the metabolomic data of the raw and salt-processed Raspberry chingii. Consistent with the PCA findings, the OPLS-DA demonstrated a clear metabolic distinction between the raw and processed samples (Figure 2A). The model evaluation parameters (R^2^X > 0.5, R^2^Y > 0.5, Q^2^ > 0.9) indicated robust goodness-of-fit and predictive reliability (Figure 2B). Student’s *t*-test confirmed significant differences (*p* < 0.05) in the metabolite concentrations, leading to the identification of 13 key plant secondary metabolites with marked variation (Table 1). The screened plant secondary metabolites were predominantly flavonoids, terpenoids, alkaloids, and phenolic compounds. Studies have demonstrated that plant-derived compounds such as flavonoids and terpenoids exert therapeutic effects by delaying glomerulosclerosis and ameliorating proteinuria through antioxidative, anti-inflammatory, metabolic regulation, and multi-pathway synergistic mechanisms. Our findings revealed that the salt-processed Raspberry chingii exhibited significantly higher levels of flavonoid and terpenoid metabolites compared to the raw material. Building on these findings, we further employed network pharmacology to systematically investigate the potential mechanisms underlying the therapeutic efficacy of salt-processed Raspberry chingii against diabetic nephropathy.

### 3.2. Network Analysis

**Figure 3:**
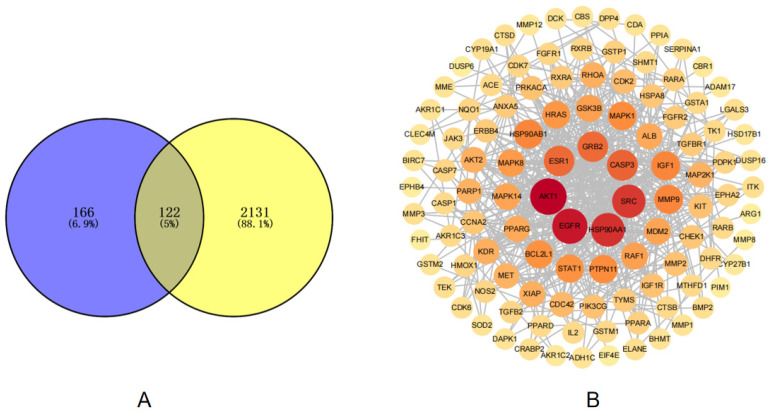
(**A**) Venn diagram (yellow: disease target; blue: component target) and (**B**) key target screening diagram.

This study employed network pharmacology to systematically investigate the therapeutic mechanisms of salt-processed Raspberry chingii against diabetic nephropathy (DN). Using Swiss Target Prediction, we identified potential targets for 13 active components, yielding 288 candidate targets. Disease-related targets associated with DN (*n* = 2253) were retrieved from the GeneCards (25 January 2025) database. A Venn diagram generated via the Weishangxin online platform (https://www.bioinformatics.com.cn/, accessed on 1 June 2025) revealed 122 intersection targets shared between the component-related and disease-related targets (Figure 3A), suggesting their critical role in mediating therapeutic effects. To investigate the interactions among the potential targets, 122 candidate targets were uploaded to the STRING platform to construct a PPI network. When using the SwissTargetPrediction database, we selected potential targets with probability scores of higher than 0.7 as candidate targets, while the predictions below this threshold were classified as secondary priority targets. For the GeneCards database, we filtered the genes based on the Relevance Score, retaining only those with Relevance Scores of greater than 0.5 to ensure that the selected targets were highly relevant to the research topic. The resulting network comprised 122 nodes and 428 edges, with each node representing a target protein. The TSV file was imported into Cytoscape 3.6.0 for visualization and topological analysis. Targets exceeding one standard deviation above the mean in Closeness Centrality (CC), Betweenness Centrality (BC), and Degree (D) were identified as key targets.

Key Targets for Diabetic Nephropathy Intervention: The top 10 targets, ranked by Degree value, were AKT1, EGFR, HSP90AA1, SRC, CASP3, GRB2, ESR1, HSP90AB1, HRAS, and GSK3B (Figure 3B). These targets may represent the core therapeutic nodes of salt-processed raspberries. These genes (e.g., AKT1, EGFR, SRC) collectively contribute to the pathological progression of diabetic nephropathy by regulating insulin signaling, inflammation, oxidative stress, apoptosis, and renal fibrosis, leading to glomerular injury, metabolic disturbances, and renal functional deterioration.

### 3.3. Analysis of GO and KEGG Results

**Figure 4:**
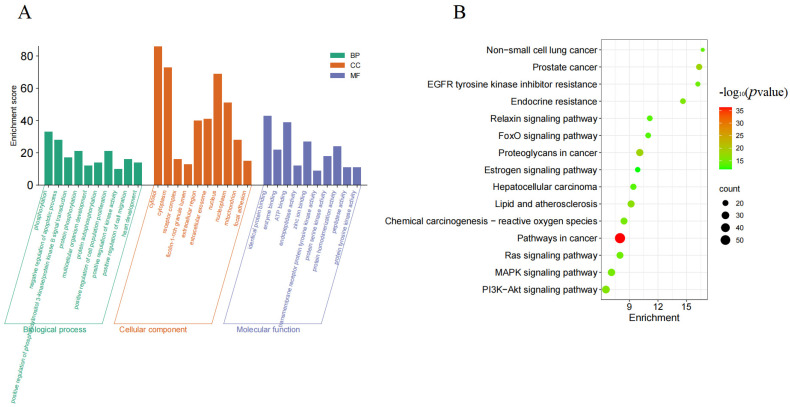
(**A**) GO enrichment analysis and (**B**) KEGG pathway analysis.

The GO enrichment analysis results from the DAVID database, filtered by *p*-values of <0.05, identified 384 GO terms, including 279 biological processes (BPs), 42 cellular components (CCs), and 63 molecular functions (MFs). The top ten BP terms and top five CC/MF terms were visualized (Figure 4A). The key BP terms involved signal transduction, regulation of transcription from the RNA polymerase II promoter (positive/negative), responses to drugs, regulation of apoptotic processes, and inflammatory responses. The CC terms were enriched in plasma membrane, cytosol, and cytoplasm. The MF terms included protein binding, ATP binding, protein homodimerization activity, and protein serine/threonine/tyrosine kinase activity. These findings highlight the multi-target therapeutic effects of salt-processed Rubi on diabetic nephropathy. The KEGG pathways enriched by the DAVID database (filtered at *p* < 0.05) were ranked by the “Count” value (number of potential gene targets in each pathway), and the top 15 pathways were selected for visualization (Figure 4B). These included non-small-cell lung cancer, prostate cancer, EGFR tyrosine kinase inhibitor resistance, endocrine resistance, the relaxin signaling pathway, the FoxO signaling pathway, proteoglycans in cancer, the estrogen signaling pathway, hepatocellular carcinoma, lipids and atherosclerosis, chemical carcinogenesis—reactive oxygen species, pathways in cancer, the Ras signaling pathway, the MAPK signaling pathway, and the PI3K-Akt signaling pathway. The results indicate that the biological processes combating diabetic nephropathy involve the regulation of apoptosis, and the MAPK signaling pathway—highlighted by KEGG enrichment—is closely associated with apoptosis. Therefore, we further validated the apoptotic pathways and MAPK signaling pathway signaling in a diabetic nephropathy cell model.

### 3.4. Effect of Salt-Processed Fupenzi on Cell Proliferation

**Figure 5:**
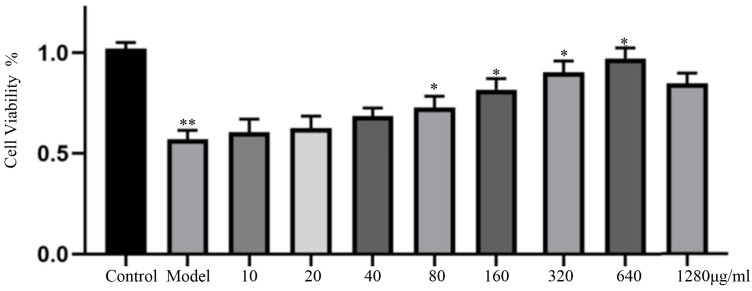
Cell value-adding rate (*n* = 6, ** *p* < 0.01, * *p* < 0.05 ).

The cell proliferation rate was calculated using this formula: Proliferation rate (%) = [(Treatment group − Blank group)/(Control group − Blank group)] × 100% (results shown in the figure). Compared with the normal group, the cells in the model group (treated with 30 mM glucose) exhibited significant damage and reduced proliferation (*p* < 0.05). In contrast, treatment with salt-processed Fupenzi at concentrations of 160 μg/mL, 320 μg/mL, and 640 μg/mL significantly enhanced the cell viability, ameliorated the cellular damage, and promoted the proliferation compared to the model group (*p* < 0.05). Consequently, these three concentrations (160, 320, and 640 μg/mL) were selected for subsequent experiments (Figure 5).

### 3.5. Effect of Salt-Processed Fupenzi on the MAPK Signaling Pathway

**Figure 6:**
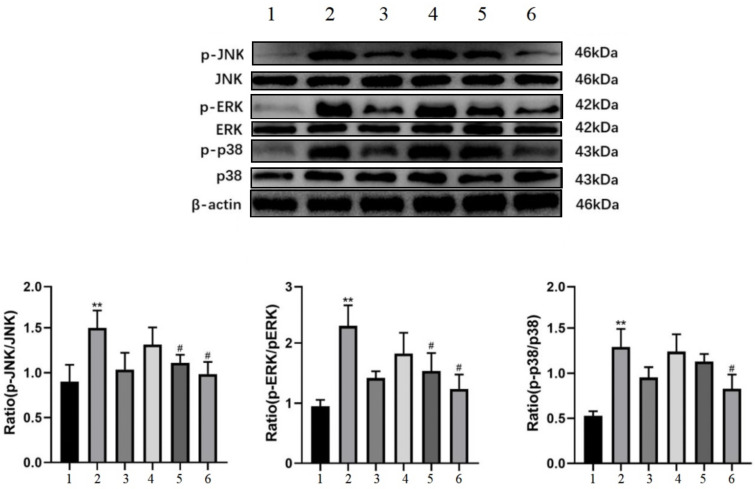
MAPK pathway-related indexes were determined by protein blotting (1. control; 2. model; 3. raspberry, 640 μg/mL; 4. salt-processed raspberry, 160 μg/mL; 5. salt-processed raspberry, 320 μg/mL; 6. salt-processed raspberry, 640 μg/mL ** *p* < 0.01, ^#^ *p* < 0.05).

To further explore the molecular mechanism of salt-processed Fupenzi in treating diabetic nephropathy (DN), Western blot analysis combined with network pharmacology enrichment results was performed to evaluate the MAPK signaling pathway (Figure 6). Compared with the control group (Con), the model group showed significantly increased phosphorylation levels of p-p38, p-ERK, and p-JNK (*p* < 0.01), as well as elevated ratios of p-p38/p38, p-ERK/ERK, and p-JNK/JNK (*p* < 0.05). The results demonstrated that the salt-processed Fupenzi effectively suppressed the phosphorylation of p38, ERK, and JNK, thereby inhibiting the activation of the MAPK pathway. Furthermore, the salt-processed Fupenzi exhibited superior inhibitory effects on p38, ERK, and JNK compared with crude (unprocessed) Fupenzi.

## 4. Discussion

The salt processing of *Raspberry* represents a critical step in enhancing its therapeutic potential, as evidenced by the marked upregulation of bioactive metabolites such as flavonoids, triterpene saponins, and phenolic acids. These compounds, particularly Kaempferol-3-O-(6″-galloyl)glucoside and Raspberryides H/F, are known for their antioxidant and anti-inflammatory properties, aligning with previous studies on salt-processed herbs where it was shown that ionic interactions during processing may stabilize or amplify specific phytochemicals [21]. Studies have shown that increases in the content of ellagic acid can improve diabetes and its complications by inhibiting inflammation, oxidative stress, hyperglycemia, apoptosis, and insulin resistance [22]. Additionally, kaempferol and its derivatives can activate AMPK and peroxisome proliferator-activated receptor α (PPARα); inhibit CCAAT enhancer-binding protein α (C/EBP-α), sterol regulatory element-binding protein 1c (SREBP1c), and PPARγ; and protect β cells [23]. Salt treatment can regulate stress resistance through the MAPK signaling pathway, including the upregulation or downregulation of enzyme or gene expression in key metabolic pathways, thereby affecting the synthesis of bioactive metabolites. However, current research has not precisely elucidated the complete molecular network of these metabolic changes. Therefore, future studies may integrate metabolomics and enzymatic analyses to systematically investigate the changes in secondary metabolites (such as polyphenols and flavonoids) in raspberries before and after salt processing, as well as the dynamic regulation of key enzyme activities. Proteomics technology can be utilized to detect modifications or expression differences in related enzymes or molecules induced during salt processing [24].

The network pharmacology predicted MAPK signaling as a potential key pathway connecting the salt-processed raspberry components and DN pathogenesis. This prediction is consistent with previous research indicating that dysregulation of the MAPK pathway, specifically hyperglycemia-induced phosphorylation of p38/ERK/JNK, plays a role in promoting apoptosis and fibrosis in diabetic renal injury. Our in vitro data showed that the salt-processed raspberry extracts exhibited stronger inhibitory effects on MAPK activation compared with the raw raspberries, suggesting that salt processing may enrich metabolites with potential kinase-targeting activities.

While our study provides valuable insights into the potential of salt-processed *Raspberry* for DN management, several limitations should be acknowledged. First and foremost, as the findings are primarily based on in vitro models, they may not fully reflect the physiological complexity of human DN. Therefore, the proposed role of salt-processed *Raspberry* in suppressing the MAPK pathway and its impact on DN progression should be interpreted with caution until further validation using in vivo animal models or clinical studies. Second, the metabolomic analysis mainly focused on annotated compounds, leaving many uncharacterized metabolites unexplored. Employing advanced structural elucidation techniques, such as nuclear magnetic resonance (NMR) spectroscopy or molecular networking approaches, could help identify novel bioactive metabolites contributing to the therapeutic effects. Lastly, the underlying mechanisms by which salt processing enriches specific metabolites, including possible contributions from ion exchange, pH changes, or thermal effects during processing, remain to be further investigated.

## 5. Conclusions

This study systematically deciphered the mechanistic basis of salt-processed raspberries in mitigating diabetic nephropathy (DN) through integrated metabolomics and network pharmacology. Salt processing significantly elevated bioactive metabolites, including flavonoids (e.g., luteolin-7-O-glucoside), triterpene saponins (Raspberryides H/F), and phenolic acids (ellagic acid), which collectively targeted the MAPK signaling pathway to suppress hyperglycemia-induced renal apoptosis and inflammation. Network pharmacology identified the AKT1, EGFR, and MAPK cascades as central therapeutic nodes, corroborated by in vitro experiments demonstrating the salt-processed raspberries’ superior inhibition of p38/ERK/JNK phosphorylation compared with unprocessed raspberries. These findings validate the traditional use of salt processing to enhance pharmacological activity and provide a molecular rationale for optimizing herbal processing techniques. However, limitations such as the reliance on cell-based models and incomplete metabolite annotation highlight the need for further translational studies and advanced analytical approaches. This work establishes a foundation for standardizing salt-processed raspberry production and advancing its application in DN management.

## Figures and Tables

**Table 1:** Compositional variation between raw raspberries and salt-processed raspberries.

Compounds	Formula	VIP	Log_2_FC	Type	Class
Ellagic acid	C_14_H_6_O_8_	1.60	2.64	up	Phenolic acids
Kaempferol-3-O-(6″-galloyl)glucoside	C_28_H_24_O_15_	1.53	1.52	up	Flavonols
Luteolin-7-O-glucoside (Cynaroside)	C_21_H_20_O_11_	1.52	1.55	up	Flavones
2,4,6-Trihydroxybenzoic acid	C_7_H_6_O_5_	1.48	1.36	up	Phenolic acids
Dihydrokaempferol-3-O-glucoside	C_21_H_22_O_11_	1.47	1.13	up	Flavanonols
Kaempferol-3-O-(6″-malonyl)glucoside	C_24_H_22_O_14_	1.36	1.16	up	Flavonols
Yenhusomine	C_21_H_23_NO_6_	1.30	1.92	up	Isoquinoline alkaloids
Raspberryide H	C_36_H_56_O_10_	1.17	1.11	up	Triterpene saponins
Raspberryide F	C_36_H_56_O_9_	1.15	1.30	up	Triterpene saponins
Palmatine	C_21_H_22_NO_4_^+^	1.12	3.50	up	Alkaloids
Geraniinic acid B	C_41_H_28_O_27_	1.11	4.28	up	Tannins
Sanguiin H1	C_34_H_26_O_22_	1.06	2.79	up	Tannins
Granatin A	C_34_H_24_O_22_	1.03	2.49	up	Tannins

## Data Availability

The data that support the findings of this study are available from the corresponding author upon reasonable request.

## Supplementary Materials

The following supporting information can be downloaded at: https://www.mdpi.com/article/10.3390/metabo15070435/s1, Figure S1: Overlay of mass control (QC) sample essential spectrum detection total ion current (TIC) graph and classification of detected metabolites; Table S1: Compositional variation between raw raspberries and salt-processed raspberries.
