# Supplementary material for: Analysis of Processing Impact on Raspberries Based on Broad-Spectrum Metabolomics
Source: Metabolites. 2025 Jun 26;15(7):435. doi: 10.3390/metabo15070435 (PMC12301035; doi:10.3390/metabo15070435)
Supplement: Supplementary file 1 [file metabolites-15-00435-s001.zip › metabolites-3666306-supplementary (2).pdf]

Table S1:Compositional variation between raw raspberries and salt-processed raspberries

	Ion mode	Q1 (Da)	Q3 (Da)	DP	CE	Compounds	Class	Formula	Level	R-1	R-2	R-3	S-1	S-2	S-3	VIP	P-value	FDR	Fold_Change	Log2FC	Type
Zbqn001288	Negative	202.11	130.09	-50	-30	(3-hydroxypropanoyl)-L-leucine	Amino acids and derivatives	$C_9H_{17}NO_4$	1	898000	1170000	510000	212000	281000	280000	1.48	0.0864	0.334	3.34	1.74	up
MWS20207	Negative	939.11	769.09	-50	-30	1,2,3,4,6-Penta-O-galloyl-β-D-glucose	Phenolic acids	$C_{41}H_{32}O_{26}$	2	235000	136000	212000	33500	93900	127000	1.19	0.0546	0.283	2.29	1.2	up
Lmsn004177	Negative	779.11	301	-50	-30	1-O-p-Coumaroyl-3-O-galloyl-4,6-(S)-HHDP-β-D-glucose	Phenolic acids	$C_{36}H_{28}O_{20}$	1	113000	60500	42900	16500	46700	41600	1.05	0.213	0.468	2.07	1.05	up
pmb1912	Positive	474.17	327.12	40	30	10-Formyltetrahydrofolic Acid	Alkaloids	$C_{20}H_{23}N_7O_7$	1	2270000	823000	1050000	502000	459000	947000	1.12	0.235	0.495	2.17	1.12	up
Jmyp005451	Positive	352.15	308.13	50	30	13-Methylpalmatrubine	Isoquinoline alkaloids	$C_{21}H_{22}NO_4^+$	1	2180	42500	44800	436	436	436	1.43	0.168	0.422	68.4	6.1	up
Wafn002491	Negative	385.08	209.03	-50	-30	2-O-Feruloylglucaric Acid	Phenolic acids	$C_{16}H_{18}O_{11}$	2	163000	166000	269000	164000	23300	104000	1.02	0.131	0.395	2.05	1.04	up
Lmqp000329	Positive	296.07	104.11	50	30	5-Aminoimidazole ribonucleotide	Nucleotides and derivatives	C8H14N3O7P	1	2340000	2610000	3000000	1230000	961000	942000	1.58	0.00541	0.147	2.54	1.34	up
Lajp004190	Positive	547.16	393.1	80	30	Afzelechin-(4α→8)-epiafzelechin	Flavanols	$C_{30}H_{26}O_{10}$	1	615000	787000	792000	319000	386000	344000	1.57	0.0149	0.189	2.09	1.07	up
pmp000100	Positive	867.21	715.2	50	30	Arecatannin B1	Proanthocyanidins	$C_{45}H_{38}O_{18}$	1	204000	214000	291000	111000	128000	103000	1.53	0.0389	0.247	2.08	1.06	up
MWSmce389	Positive	209.12	179.07	50	30	Beta-asarone	Others	$C_{12}H_{16}O_3$	1	353000	146000	134000	123000	61600	83400	1.19	0.225	0.486	2.36	1.24	up

Cmhn001	Negat	935.	633.							540	72600	70600	37400	21400	25900	1.	0.008	0.1			
611	ive	09	08	-50	-30	Casuarictin	Tannin	$C_{41}H_{28}O_{26}$	1	000	0	0	0	0	0	49	75	55	2.33	1.22	up
Lmsn001	Negat	935.								668	93000	77300	30200	29100	33500	1.	0.021	0.2			
955	ive	08	301	-50	-30	Casuarinin	Tannin	$C_{41}H_{28}O_{26}$	1	000	0	0	0	0	0	59	7	06	2.55	1.35	up
Lmxn000	Negat	341.	89.0							256	24400	21200		15800	10200	1.	0.011	0.1			
398	ive	11	2	-50	-30	D-Lactose*	Saccharides	$C_{12}H_{22}O_{11}$	1	000	0	0	94200	0	0	47	1	66	2.01	1.01	up
Lmsn000	Negat	341.	89.0							252	32200	27300	14400	13100	11700	1.	0.010	0.1			
381	ive	112	22	-50	-30	D-Maltose*	Saccharides	$C_{12}H_{22}O_{11}$	1	000	0	0	0	0	0	58	1	6	2.16	1.11	up
	Negat	181.	71.0							545	21700	30200	11400	17800	13900	1.		0.4			
mws0214	ive	07	1	-40	-30	D-Sorbitol	Saccharides	$C_6H_{14}O_6$	1	000	0	0	0	0	0	32	0.161	22	2.47	1.3	up
	Negat	341.	119.							173	13400	21300				1.	0.037	0.2			
pme0519	ive	11	03	-80	-20	D-Sucrose*	Saccharides	$C_{12}H_{22}O_{11}$	1	000	0	0	62800	70200	89500	51	2	43	2.33	1.22	up
										114											
Lazn006	Negat	585.	537.							000	91500	97400	34100	51200	47700	1.	0.003	0.1			
001	ive	23	21	-80	-30	Dihydrobuddlenol B	Lignans	$C_{31}H_{38}O_{11}$	1	0	0	0	0	0	0	54	27	29	2.28	1.19	up
Lmlp005	Positi	451.	289.							426	28400	38100	16300	12800	20700	1.	0.024	0.2			
236	ve	12	08	50	30	Dihydrokaempferol-3-O-glucoside*	Flavanonols	$C_{21}H_{22}O_{11}$	2	000	0	0	0	0	0	48	3	17	2.19	1.13	up
	Negat	181.	101.							721	40000	46200	21400	31200	21900	1.		0.3			
pme2237	ive	072	022	-40	-20	Dulcitol*	Saccharides	$C_6H_{14}O_6$	1	000	0	0	0	0	0	4	0.093	42	2.12	1.09	up
Lwhp010	Positi	160.	115.				Quinoline			464						1.	0.039	0.2			
633	ve	08	05	80	30	Echinopsine	alkaloids	$C_{10}H_9NO$	2	00	82900	75200	26700	30100	13600	42	9	47	2.9	1.54	up
	Negat	341.	179.							677	28700	30300	13500	13400	12700	1.		0.4			
mws1080	ive	11	06	-80	-20	Galactinol	Saccharides	$C_{12}H_{22}O_{11}$	1	000	0	0	0	0	0	44	0.15	16	3.2	1.68	up
MWS202	Negat	951.	301.							963						1.		0.1			
05	ive	072	002	-50	-30	Geraniin	Tannin	$C_{41}H_{28}O_{27}$	2	00	83800	90500	9340	9340	9340	63	0.002	29	9.65	3.27	up
Hmln001	Negat	951.	907.	-50	-30	Geraniinic acid B	Tannin	$C_{41}H_{28}O_{27}$	2	754	65300	87900	55800	28800	33300	1.	0.007	0.1	19.4	4.28	up

763	ive	08	09							000	00	00	0	0	0	61	28	47			
										0											
										112											
Lmsn001	Negat	783.								000	16900	23000	18100	92000	95100	1.	0.080	0.3			
369	ive	07	301	-50	-30	Granatin A	Tannin	$C_{34}H_{24}O_{22}$	1	0	00	00	0	0	0	11	4	24	2.49	1.32	up
	Negat	341.	89.0							279	28700	28500	10600	14500	14300	1.	0.005	0.1			
mws5038	ive	114	24	-50	-30	Isomaltulose*	Saccharides	$C_{12}H_{22}O_{11}$	1	000	0	0	0	0	0	57	49	47	2.17	1.11	up
										390											
Lmmn00	Negat	489.	285.							000	71000	45900	21300	13000	37900	1.	0.088	0.3			
3398	ive	11	04	-50	-30	Kaempferol-3-O-(6''-O-acetyl)glucoside	Flavonols	$C_{23}H_{22}O_{12}$	1	0	00	00	00	00	00	24	8	39	2.16	1.11	up
Lmyp003	Positi	601.	287.							759	10700					1.	0.013	0.1			
599	ve	124	064	50	30	Kaempferol-3-O-(6''-galloyl)glucoside*	Flavonols	$C_{28}H_{24}O_{15}$	1	00	0	80300	28300	23200	40100	53	1	81	2.87	1.52	up
										399											
Lmmp00	Positi	535.	287.			Kaempferol-3-O-(6''-malonyl)glucoside				000	74000	69900	37100	21100	23800	1.	0.069	0.3			
3817	ve	114	054	50	30	*	Flavonols	$C_{24}H_{22}O_{14}$	1	0	00	00	00	00	00	37	5	04	2.24	1.16	up
										383											
	Positi	581.	287.							000	44800	84900	13100	17200	37500	1.		0.4			
HJAP148	ve	15	06	50	30	Kaempferol-3-O-sambubioside	Flavonols	$C_{26}H_{28}O_{15}$	1	0	00	00	00	00	00	25	0.134	01	2.48	1.31	up
										849											
Lmqp003	Positi	581.	287.			Kaempferol-3-O-xylosyl(1→2)glucosid				000	54400	87300	17200	17200	11600	1.	0.026	0.2			
887	ve	15	05	50	30	e	Flavonols	$C_{26}H_{28}O_{15}$	1	0	00	00	00	00	00	58	7	26	4.93	2.3	up
										783											
pmp0005	Negat	442.	263.							000	60700	79600	31600	22300	23200	1.	0.006	0.1			
87	ive	38	14	-50	-30	Luteolin-7-O-(6''-malonyl)glucoside*	Flavones	$C_{24}H_{22}O_{14}$	1	0	00	00	00	00	00	57	96	47	2.83	1.5	up
MWSHY	Negat	448.	2654							956	92100	67400	24100	23700	39300	1.	0.010	0.1			
0104	ive	36	.84	-50	-30	Luteolin-7-O-glucoside (Cynaroside)*	Flavones	$C_{21}H_{20}O_{11}$	1	000	000	000	000	000	000	53	1	6	2.93	1.55	up

										00											
Hmdn001963	Negative	234.24	120.65	-50	-30	Methyl Brevifolincarboxylate	Organic acids	$C_{14}H_{10}O_8$	1	2400000	1740000	1120000	598000	333000	385000	1.5	0.0639	0.297	4	2	up
pmn001623	Positive	274.25	110.32	-50	-30	Methyl neochebulagate	Tannin	$C_{42}H_{34}O_{28}$	2	78400	97700	69300	23700	28800	22200	1.59	0.0167	0.192	3.28	1.72	up
Wayn000341	Positive	276.29	143.69	-40	-30	N-(1-Deoxy-1-fructosyl)Asparagine	Amino acids and derivatives	$C_{10}H_{18}N_2O_8$	1	6350000	6040000	6180000	2470000	2560000	2670000	1.62	0.0000201	0.0174	2.41	1.27	up
Wayn002083	Negative	305.32	241.35	-80	-20	N-(1-Deoxy-1-fructosyl)Phenylalanine	Amino acids and derivatives	$C_{15}H_{21}NO_7$	1	9050000	9300000	7530000	3110000	3830000	4240000	1.57	0.00343	0.129	2.31	1.21	up
Wayp001024	Positive	291.32	125.54	-80	-30	N-(1-Deoxy-1-fructosyl)Valine	Amino acids and derivatives	$C_{11}H_{21}NO_7$	1	2920000	2980000	8530000	1420000	1440000	1650000	1.34	0.217	0.476	3.2	1.68	up
Smcp000882	Positive	327.36	180.4	50	30	N-benzoyl-2-aminoethyl-β-D-glucopyranoside	Alkaloids	$C_{15}H_{21}NO_7$	1	2970000	2770000	2510000	1220000	1550000	1140000	1.57	0.00138	0.129	2.11	1.07	up
Lcsn000653	Positive	667.66	400.69	-40	-20	Niga-ichigoside F3	Triterpene Saponin	$C_{38}H_{60}O_{12}$	1	4700000	1590000	2560000	1580000	1400000	602000	1.16	0.189	0.443	2.46	1.3	up
MWSmce078	Positive	342.36	150.12	80	30	Palmatine	Isoquinoline alkaloids	$C_{21}H_{22}NO_{4}^{+}$	2	8360	157000	214000	14300	17900	1490	1.05	0.2	0.457	11.3	3.5	up
Lmjp102213	Negative	564.55	300.47	-80	-20	Pedunculagin	Tannin	$C_{34}H_{24}O_{22}$	1	393000	443000	364000	174000	112000	150000	1.57	0.0012	0.129	2.76	1.46	up
Lmsn005	Negat	610.	300.	-50	-30	Phloretin-4'-O-(6"-Galloyl)glucoside	Chalcones	$C_{28}H_{28}O_{14}$	2	203	24200	24800	8860	11300	12100	1.	0.003	0.1	2.15	1.1	up

788	ive	55	46							00						56	05	29			
Lcsp0006	Negat	538.	200.				Triterpene			356						1.		0.4			
67	ive	51	46	80	30	Rubuside H	Saponin	$C_{36}H_{56}O_{10}$	2	00	14100	19400	15300	10100	6690	17	0.187	41	2.16	1.11	up
Hmqn00	Negat	740.	380.							422	59000	73400				1.	0.031	0.2			
3158	ive	62	47	-80	-20	Sanguiin H1*	Tannin	$C_{34}H_{26}O_{22}$	1	000	0	0	84400	84400	84400	6	3	31	6.89	2.79	up
										440											
Lczn000	Negat	1110	470.							000	46400	37300	23900	15300	19000	1.	0.003	0.1			
070	ive	.92	91	-50	-30	Sanguiin H7	Tannin	$C_{34}H_{26}O_{23}$	2	0	00	00	00	00	00	53	36	29	2.2	1.14	up
	Negat	176.	50.6							519	61100	53900	12400	17300		1.	0.000	0.0			
pme2024	ive	24	9	-50	-30	Serotonin; 5-Hydroxytryptamine	Plumerane	$C_{10}H_{12}N_2O$	2	000	0	0	0	0	80100	56	377	659	4.43	2.15	up
Cmhn000	Negat	484.	300.					C27H22O1		406	53700	43900	24600	15900	20800	1.	0.008	0.1			
855	ive	46	52	-50	-30	Valoneoyl-D-glucose	Tannin	9	1	000	0	0	0	0	0	53	54	54	2.26	1.17	up
Jmyp004	Positi	476.	145.				Isoquinoline			594						1.		0.2			
117	ve	51	54	-50	-30	Yenhusomine	alkaloids	$C_{21}H_{23}NO_6$	2	00	44100	75800	5380	5380	36400	3	0.034	38	3.8	1.93	up
MWSmc	Negat	194.	80.6							371	11600	17000	16000			1.		0.5			
e482	ive	25	5	-50	-30	alpha-Asarone	Phenolic acids	$C_{12}H_{16}O_3$	1	000	0	0	0	71600	59900	07	0.253	14	2.26	1.17	up
										207											
Lcsn0006	Positi	649.	433.			ent-3α,16β,17,19-Tetrahydroxykauran-1				000	22600	16600	86900	82700	10800	1.	0.014	0.1			
48	ve	68	92	50	30	9-ylacetate-17-O-β-D-glucopyranoside	Ditepenoids	$C_{28}H_{46}O_{10}$	1	0	00	00	0	0	00	55	4	86	2.16	1.11	up
Wafn003	Negat	388.	184.			1-O-Acetyl-Glucopyranose				373						1.		0.4			do
633	ive	49	54	50	30	6-Hydroxydecanoate	Saccharides	$C_{18}H_{32}O_9$	2	00	5310	5310	33400	26500	88900	13	0.228	88	0.322	-1.64	wn
Wcfn002	Negat	222.	101.			2,3-Dihydroxy-1-(4'-hydroxy-3'-methox				329	26100	31900	36400	60500	86900	1.		0.4			do
460	ive	23	35	50	30	yphenyl)-propan-1-one	Others	$C_{10}H_{12}O_5$	1	000	0	0	0	0	0	27	0.165	22	0.495	-1.02	wn
Xmbp00	Negat	182.	182.							345	67500	60800	14600	15000	12200	1.	0.003	0.1			do
1692	ive	17	47	50	30	2,4,6-Trihydroxybenzoic acid	Phenolic acids	$C_7H_6O_5$	2	000	0	0	00	00	00	48	28	29	0.389	-1.36	wn
Zbln0061	Negat	360.	284.	-50	-30	3,3'-O-Dimethylellagic Acid	Tannin	$C_{16}H_{10}O_8$	1	128	12800	12800	63900	71200	10500	1.	0.034	0.2	0.159	-2.65	do

02	ive	34	62							00					0	6	1	38			wn
Zjbn1025	Negat	304.	156.			3,7-dihydroxy-2,4-dimethoxyphenanthr				390			12100			1.	0.063	0.2			do
29	ive	35	51	-50	-30	ene	Others	$C_{16}H_{14}O_4$	1	00	48200	36500	0	75500	80800	49	6	97	0.446	-1.17	wn
Lafp0027	Negat	386.	110.			3-Hydroxy-1-(4-hydroxy-3,5-dimethox				129						1.	0.075	0.3			do
22	ive	43	64	-50	-30	y-phenyl)-propan-1-one glucoside	Phenolic acids	$C_{17}H_{24}O_{10}$	2	0	1290	1290	6440	10700	16400	58	4	18	0.115	-3.12	wn
										112											
	Positi	143.	84.2							000	10400	74700	16800	16100	41000	1.		0.4			do
pmb0764	ve	2	3	-50	-30	4-Methyl-5-thiazoleethanol	Others	C_6H_9NOS	2	0	00	0	00	00	00	29	0.208	64	0.393	-1.35	wn
MWS201	Positi	126.	72.3				Aldehyde			187	13100	27700	12300	27700	16100	1.	0.067	0.3			do
72	ve	11	4	-40	-30	5-Hydroxymethylfurfural*	compounds	$C_6H_6O_3$	2	000	0	0	00	00	00	56	9	01	0.106	-3.24	wn
MWSHC	Positi	154.	60.3				Aldehyde			195	13900	39200	13800	14300	15800	1.	0.000	0.0			do
20158	ve	15	5	-50	-30	5-Methoxyfurfural*	compounds	$C_6H_6O_3$	2	000	0	0	00	00	00	55	324	659	0.165	-2.6	wn
										512											
Wcsn010	Negat	274.	148.				Free fatty			000	80500	50200	43600	26000	94200	1.		0.4			do
524	ive	44	51	-50	-30	8,11-Heptadecadienoic acid	acids	$C_{17}H_{30}O_2$	1	0	00	00	000	000	00	32	0.175	33	0.23	-2.12	wn
Cmsn006	Negat	236.	128.			9,10-Dihydro-2-methoxy-4,5-phenanthr	PhenAnthraqu			214	25700	49100	63500	82400	77500	1.	0.019	0.2			do
847	ive	26	51	-50	-30	enediol	inones	$C_{15}H_{14}O_3$	1	000	0	0	0	0	0	4	5	06	0.431	-1.22	wn
							Amino acids														
MWStz2	Negat	208.	112.				and			118			14100	18300	34300	1.		0.4			do
05	ive	25	35	-50	-30	Cyclo(D-Phe-L-Pro)*	derivatives	$C_{14}H_{16}N_2O_2$	1	000	97900	86200	0	0	0	3	0.184	36	0.452	-1.14	wn
							Amino acids														
MWStz0	Positi	204.	120.				and			212			11700		14400	1.	0.014	0.1			do
83	ve	25	65	-40	-30	Cyclo(D-Val-L-Pro)	derivatives	$C_{10}H_{16}N_2O_2$	2	00	41400	20200	0	92400	0	53	3	86	0.234	-2.09	wn
							Amino acids														
MWStz0	Positi	186.	113.				and			101	13600	10300	28300	20900	21200	1.	0.023	0.2			do
91	ve	23	25	-80	-20	Cyclo(L-Ala-L-Pro)	derivatives	$C_8H_{12}N_2O_2$	1	000	0	0	0	0	0	52	2	11	0.483	-1.05	wn

Lmrj002698	Negative	172.22	114.67	-80	-30	Cyclo(Pro-Val)	Amino acids and derivatives	$C_{10}H_{16}N_2O_2$	2	26600	21200	20200	104000	77500	128000	1.59	0.0294	0.229	0.22	-2.19	down
Hmlp001371	Positive	195.23	111.34	50	30	Cyclo(Tyr-Ala)	Amino acids and derivatives	$C_{12}H_{14}N_2O_3$	1	28800	25900	26600	87200	73400	79900	1.62	0.00407	0.147	0.338	-1.56	down
Zaxn006641	Positive	302.46	204.67	-40	-20	Dehydroabietic acid	Ditepenoids	$C_{20}H_{28}O_2$	2	117000	165000	74900	985000	714000	191000	1.31	0.158	0.422	0.189	-2.41	down
Wcop008876	Positive	288.44	198.65	80	30	Ent-Kaurenal	Ditepenoids	$C_{20}H_{30}O$	2	159000	94400	182000	306000	274000	356000	1.43	0.00957	0.159	0.466	-1.1	down
MWSmce177	Positive	194.2	80.32	-80	-20	Ethyl ferulate	Phenolic acids	C12H14O4	1	7080	17100	6320	19100	61600	11400	1.03	0.317	0.577	0.331	-1.59	down
pme1109	Negative	151.13	79.58	-50	-30	Guanine	Nucleotides and derivatives	$C_5H_5N_5O$	1	125000	163000	154000	603000	713000	677000	1.61	0.00168	0.129	0.221	-2.18	down
Lmmn002274	Negative	592.6	300.59	80	30	Isolariciresinol-9'-O-glucoside*	Lignans	$C_{26}H_{34}O_{11}$	2	884000	431000	594000	802000	1710000	1340000	1.23	0.117	0.375	0.495	-1.01	down
MWSmce040	Negative	152.15	166.45	-80	-20	Isovanillin*	Aldehyde compounds	$C_8H_8O_3$	1	2250000	3230000	1750000	5490000	3370000	6330000	1.33	0.0761	0.318	0.476	-1.07	down
Lmyp004407	Negative	525.48	300.36	-50	-30	Kaempferol-3-O-(2''-p-Coumaroyl)galactoside*	Flavonols	$C_{30}H_{26}O_{13}$	1	5390000	13700000	10000000	20100000	18400000	21500000	1.33	0.0373	0.243	0.486	-1.04	down
Lmhp008589	Negative	520.54	300.28	-50	-30	LysoPE 18:3(2n isomer)	LPE	$C_{23}H_{42}NO_7P$	1	1510000	1970000	2190000	3690000	3800000	4390000	1.54	0.00225	0.129	0.478	-1.07	down

Lssp2100<br>87	Negat<br>ive	370.<br>42	300.<br>17	-50	-30	Matairesinol	Lignans	$C_{20}H_{22}O_6$	2	817<br>000	95300<br>0	62200<br>0	20300<br>00	27500<br>00	93800<br>0	1.<br>23	0.167	0.4<br>22	0.419	-1.26	do<br>wn
MWS201<br>458	Negat<br>ive	210.<br>25	150.<br>32	-50	-30	Phe-Ile	Amino acids<br>and<br>derivatives	$C_{15}H_{22}N_2O_3$	1	210<br>000	50900<br>0	28400<br>0	73300<br>0	71400<br>0	57300<br>0	1.<br>32	0.043<br>1	0.2<br>52	0.496	-1.01	do<br>wn
NK10264<br>324	Positi<br>ve	126.<br>11	71.4<br>5	-50	-30	Phloroglucinol; 1,3,5-Benzenetriol	Others	$C_6H_6O_3$	2	204<br>000	17500<br>0	33900<br>0	15100<br>00	17200<br>00	16100<br>00	1.<br>59	0.000<br>0772	0.0<br>334	0.148	-2.75	do<br>wn
Hajp0035<br>46	Negat<br>ive	464.<br>46	300.<br>26	50	30	catechin-3-O-α-L-rhamnopyranoside	Flavanols	$C_{21}H_{24}O_{10}$	2	387<br>000	52400<br>0	42500<br>0	14200<br>00	87400<br>0	83800<br>0	1.<br>48	0.079<br>3	0.3<br>21	0.427	-1.23	do<br>wn
Wmmp0<br>00175	Positi<br>ve	594.<br>44	280.<br>36	50	30	pterocaryaninB	Tannin	$C_{27}H_{22}O_{18}$	1	153<br>000	12700<br>0	12300<br>0	43300<br>0	25200<br>0	27600<br>0	1.<br>49	0.078<br>2	0.3<br>18	0.419	-1.25	do<br>wn
Lmbn002<br>648	Negat<br>ive	166.<br>17	80.2<br>4	50	30	α-Hydroxycinnamic Acid*	Phenolic acids	$C_9H_8O_3$	1	274<br>000<br>0	31200<br>00	38100<br>00	12100<br>000	48900<br>00	45000<br>00	1.<br>18	0.251	0.5<br>13	0.449	-1.16	do<br>wn
