# Supplementary figures and images for: Analysis of Processing Impact on Raspberries Based on Broad-Spectrum Metabolomics
Source: Metabolites. 2025 Jun 26;15(7):435. doi: 10.3390/metabo15070435 (PMC12301035; doi:10.3390/metabo15070435)

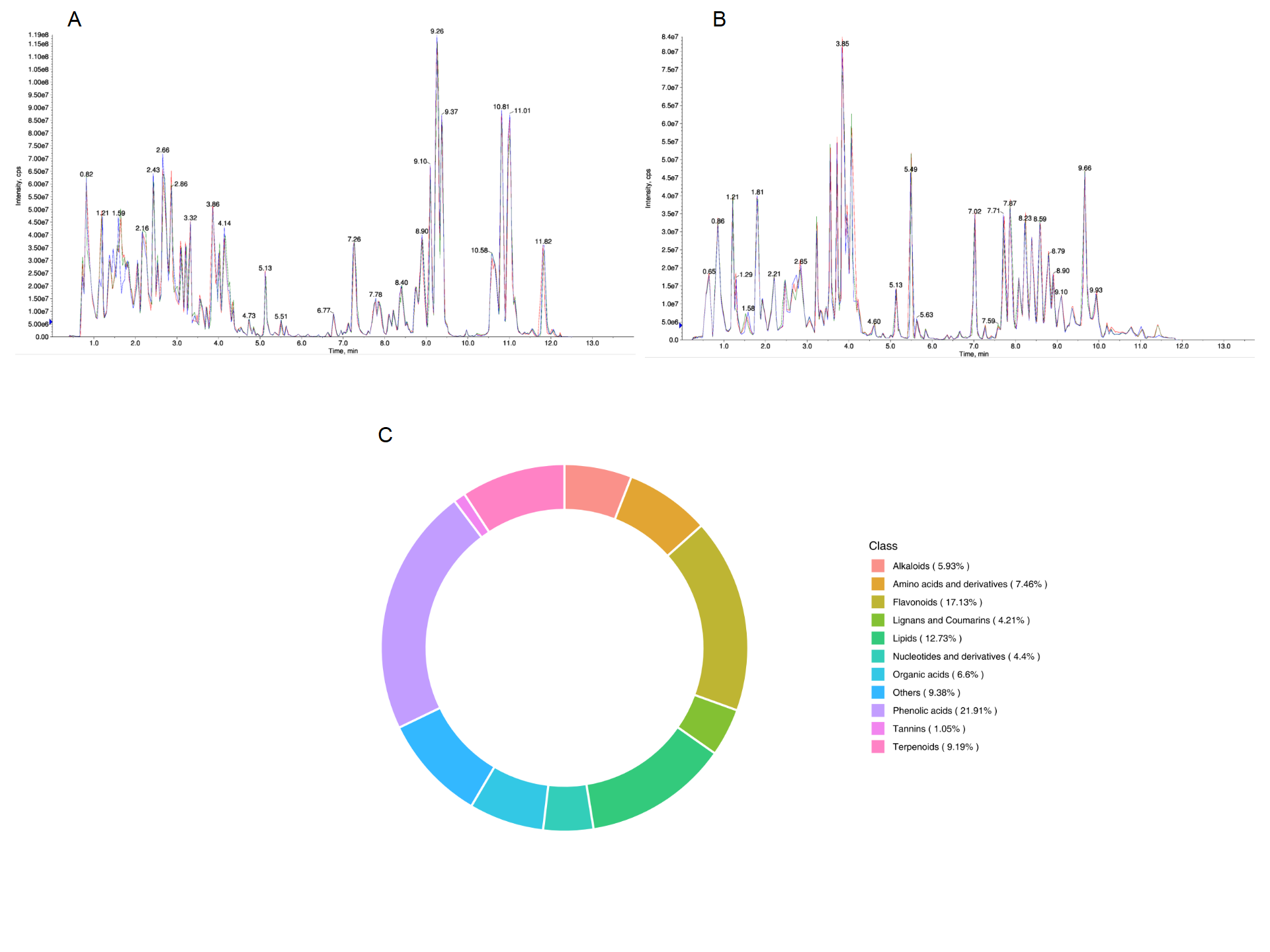

Supplement: Supplementary file 1 [file metabolites-15-00435-s001.zip › metabolites-3666306-supplementary Figure S1.png]
